# Multicolor high-resolution whole-brain imaging for acquiring and comparing the brain-wide distributions of type-specific and projection-specific neurons with anatomical annotation in the same brain

**DOI:** 10.3389/fnins.2022.1033880

**Published:** 2022-10-06

**Authors:** Zhangheng Ding, Jiangjiang Zhao, Tianpeng Luo, Bolin Lu, Xiaoyu Zhang, Siqi Chen, Anan Li, Xueyan Jia, Jianmin Zhang, Wu Chen, Jianwei Chen, Qingtao Sun, Xiangning Li, Hui Gong, Jing Yuan

**Affiliations:** ^1^Britton Chance Center and MoE Key Laboratory for Biomedical Photonics, Wuhan National Laboratory for Optoelectronics, Huazhong University of Science and Technology, Wuhan, China; ^2^Research Unit of Multimodal Cross Scale Neural Signal Detection and Imaging, HUST-Suzhou Institute for Brainsmatics, Chinese Academy of Medical Sciences, Suzhou, China

**Keywords:** multicolor, whole-brain optical imaging, real-time counterstaining, neural circuit analysis, brain-wide distribution, cell type

## Abstract

Visualizing the relationships and interactions among different biological components in the whole brain is crucial to our understanding of brain structures and functions. However, an automatic multicolor whole-brain imaging technique is still lacking. Here, we developed a multicolor wide-field large-volume tomography (multicolor WVT) to simultaneously acquire fluorescent signals in blue, green, and red channels in the whole brain. To facilitate the segmentation of brain regions and anatomical annotation, we used 4′, 6-diamidino-2-phenylindole (DAPI) to provide cytoarchitecture through real-time counterstaining. We optimized the imaging planes and modes of three channels to overcome the axial chromatic aberration of the illumination path and avoid the crosstalk from DAPI to the green channel without the modification of system configuration. We also developed an automatic contour recognition algorithm based on DAPI-staining cytoarchitecture to shorten data acquisition time and reduce data redundancy. To demonstrate the potential of our system in deciphering the relationship of the multiple components of neural circuits, we acquired and quantified the brain-wide distributions of cholinergic neurons and input of ventral Caudoputamen (CP) with the anatomical annotation in the same brain. We further identified the cholinergic type of upstream neurons projecting to CP through the triple-color collocated analysis and quantified its proportions in the two brain-wide distributions. Both accounted for 0.22%, implying CP might be modulated by non-cholinergic neurons. Our method provides a new research tool for studying the different biological components in the same organ and potentially facilitates the understanding of the processing mechanism of neural circuits and other biological activities.

## Introduction

Multicolor fluorescence microscopy has become an essential tool in neuroscience to spectrally distinguish different types of neurons and analyze their interactions. The strategy of using different fluorescent proteins to label various molecules or neurons in the same brain has been increasingly used to analyze the structure and function of neural circuits (Livet et al., 2007; Sun et al., 2019). However, due to the lack of continuous high-resolution multicolor fluorescence brain-wide optical imaging techniques, these studies have been limited to the rough studies of the whole-brain by discrete sectioning and imaging (Watabe-Uchida et al., 2012; Guo et al., 2015). Recently, several high-resolution whole-brain imaging technologies have been developed (Li et al., 2010; Gong et al., 2013, 2016; Zheng et al., 2013; Economo et al., 2016; Zhang et al., 2017; Ning et al., 2020; Zhong et al., 2021), while they only achieved single- or dual-color whole-brain imaging. There are some technical considerations. Blue DAPI-staining is widely used to provide cytoarchitecture since green and red fluorescent proteins are brighter and more common than blue ones to label interesting targets. However, DAPI imaging in the blue channel suffers axial chromatic aberration and generates crosstalk in the green channel. To overcome these challenges, alternating multicolor imaging is adapted in commercial microscopes or low-resolution whole-brain imaging (Seiriki et al., 2017). While, it is not allowed in high-resolution whole-brain imaging due to a linear increase in the imaging time of several days, and simultaneous multicolor whole-brain imaging is preferred. In this case, we might have to be concerned about chromatic aberration and spectral mixing in different channels.

Here, we developed a multicolor high-resolution whole-brain imaging to acquire the brain-wide distributions of type-specific and projection-specific neurons with anatomical annotation in the same brain and allow us to further quantitatively analyze and compare their relationship. We optimized the imaging scheme to achieve simultaneous multicolor imaging without modifying the original system configuration to overcome axial chromatic aberration and DAPI-staining crosstalk. We also corrected lateral chromatic aberration to achieve less than 1 pixel co-located error in the full FOV among three channels. To reduce data redundancy and then shorten data acquisition time, we developed a real-time automatic contour recognition and imaging region modification method using the uniform and clear DAPI signal during whole-brain imaging. To demonstrate our application potential for neural circuit study, we compared the input of cholinergic neurons and non-cholinergic neurons in CP and identified the molecular phenotype of their upstream neurons through the triple-color collocated analysis without further immunohistochemical staining. The whole brain sample was imaged at a voxel size of 0.32 × 0.32 × 2 μm^3^ for about 3 days. Our multicolor WVT will become a powerful method for the research of the input and output of the neural circuit and their relationship at the whole-brain level.

## Results

### Optimization of multicolor imaging scheme for overcoming axial chromatic aberration and spectral mixing

**FIGURE 1:**
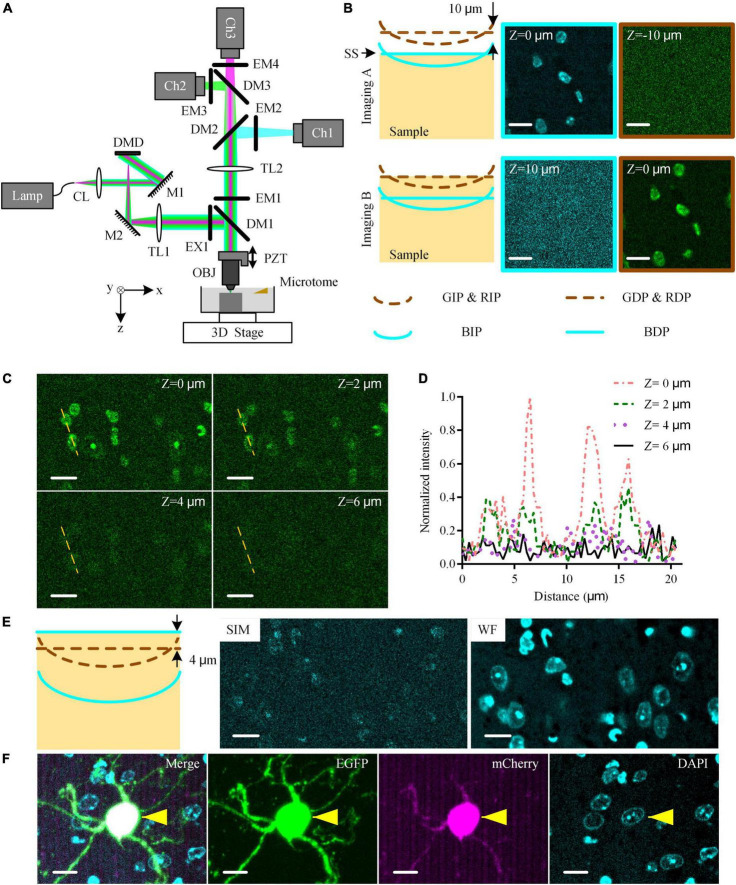
Multicolor WVT imaging method. **(A)** Schematic diagram of multicolor WVT system configuration. CL, collimating lens; M, mirror; DMD, digital micro-mirror device; TL, tube lens; EX, excite filter; DM, dichroic mirror; PZT, piezoelectric translational stage; OBJ, objective; EM, emission filter; Ch1, blue channel; Ch2, green channel; Ch3, red channel. **(B)** Imaging C57BL/6J brain tissue with DAPI real-time counterstaining when the detection plane of each channel was conjugated to the corresponding illumination plane. The sample surface coincided with the blue detection planes and green/red detection planes in Imaging **(A,B)**, respectively. All images were acquired at the edge of the FOV. The distance between the illumination planes of blue and green/red channels due to the axial chromatic aberration was 10 μm by experimental measurement. The illumination planes were curved due to field curvature. BIP/GIP/RIP, Blue/Green/Red illumination plane; BDP/GDP/RDP, Blue/Green/Red detection plane; SS, sample surface. Z = 0 μm represented the top surface of the sample. **(C)** DAPI-staining crosstalk signals in the green channel at different imaging depths with 1 μg⋅ml^–1^ DAPI solution concentration after 30 s staining time. All images were acquired at the edge of the FOV. **(D)** Intensity profiles along yellow dotted lines in **(C)**. **(E)** Schematic diagram of the optimized multicolor imaging schemes and the typical results of SIM-reconstructed image, and WF-reconstructed image of DAPI signal in the blue channel in this case. **(F)** Multicolor simultaneous imaging of EGFP and mCherry dual-labeled mouse brain tissue block with DAPI real-time counterstaining. Cyan/green/magenta images were acquired in blue/green/red channels, respectively. The “Merge” image was generated by merging cyan/green/magenta images. Yellows arrows indicated the co-located neuron. Scales bars of all images were 10 μm.

We modified the original dual-color high-resolution WVT (Gong et al., 2016) into the multicolor whole-brain imaging system (Figure 1A). A 3-band filter set (EX1, DM1, and EM1 in Figure 1A) was upgraded to filter the illumination light from a broad-spectrum mercury lamp and generate the concentric simultaneous blue/green/red excitation. An additional camera was added to realize the simultaneous detection of three channels. We used DAPI instead of propidium iodide (PI) to perform real-time counterstaining (Gong et al., 2016) during the whole-brain imaging and used other channels for the interesting fluorescent labels.

The upgraded multicolor WVT added the detection capability of the blue band, but also suffered two challenges (Figure 1B). The axial chromatic aberration of the illumination path led to the separation between the blue illumination focal plane (BIP) and green/red illumination focal plane (GIP/RIP. We omit RIP for simplicity in the following.). The emission spectrum of DAPI covering both the blue and green detection band possibly affects the detection of weak green signals. To assess these side effects on the system, we conducted blue-green dual-color imaging on a resin-embedded 8-week-old C57BL/6J male mouse brain with real-time counterstaining of DAPI (1 μg⋅ml^–1^ solution concentration, 30 s real-time staining time. Unless otherwise specified, the following is the same.). According to the principle of optical-sectioning structured illumination microscopy (OS-SIM, hereinafter referred to as SIM), the detection planes (DP) were conjugated with the illumination plane (IP) in each channel (Both Imaging A and Imaging B schematic diagrams in Figure 1B). We adjusted the position of the sample surface (SS) to coincide with the blue and green/red detection plane (BDP and GDP/RDP. We omit RDP for simplicity in the following) in turn during imaging (Imaging A and Imaging B in Figure 1B, respectively) and generated the corresponding SIM-reconstructed images. We observed the DAPI signal in the blue channel in Imaging A and its crosstalk signal in the green channel in Imaging B, respectively. We also found that the crosstalk signal was more obvious near the edge of the FOV while there was almost no crosstalk signal in the center of the FOV (Supplementary Figure 1). The reason was that the field curvature of the BIP resulted in a higher modulation contrast at the edge of the FOV than the one at the center of the FOV. The BIP was at 10 μm below the GIP due to the axial chromatic aberration determined by measuring the sample axial distance between Imaging A and Imaging B. The DAPI staining cannot cover such a depth range below the sample surface in the time window of real-time staining due to slow penetration speed. While the optical sectioning of SIM results in the depth of field of the three channels being smaller than the distance between the illumination planes. Therefore, the optically sectioned images of green and red fluorescent labels and co-located blue cytoarchitecture cannot be obtained simultaneously in the original three-color SIM imaging scheme.

To eliminate the crosstalk signal of DAPI in the green channel, we further studied the relationship between it and the imaging depth (Figures 1C,D). We found that the crosstalk signal intensity gradually decreased with increasing depth and attenuated to close to the noise intensity with the imaging depth ≥ 4 μm (Figure 1D), resulting from the limited staining depth of DAPI in real-time counterstaining (Supplementary Figure 2). Therefore, we kept the GDP/RDP always 4 μm below SS during the whole-brain imaging to avoid the DAPI-stained crosstalk signal (Schematic diagram in Figure 1E). However, the BDP, in this case, was 14 μm below SS, where there was no DAPI-staining signal. To solve this problem, we broke the conjugate relationship between the BDP and BIP and adjusted the BDP to SS, where the DAPI-staining signal was the strongest, by moving the camera of the blue channel along the optical axis. Then, the DAPI-staining at BDP was weakly modulated by structured illumination due to the separation of the BIP and BDP, resulting in an extremely low signal-to-noise ratio (SNR) in the SIM-reconstructed image (“SIM” image Figure 1E). In contrast, we reconstructed the widefield (WF) images by superposing three raw images (“WF” image in Figure 1E). The DAPI-staining soma was clearly distinguished in the WF-reconstructed image, indicating that WF reconstruction in the blue channel was competent for cytoarchitectural analysis.

Considering the effects of the above factors, we optimized the triple-color imaging scheme for whole-brain imaging. We moved the BDP 4 μm higher than GDP while keeping the IP and DP of the green/red channel conjugated. During the whole-brain imaging, we moved SS to coincide with BDP while the GDP was 4 μm below the SS. For image reconstruction, we used the SIM algorithm for the green and red channels, and WF reconstruction for the blue channel. To demonstrate the ability to detect specific fluorescence-labeled neurons and their co-located cytoarchitecture, we imaged an resin-embedded EGFP- and mCherry-labeled mouse brain tissue block (Figure 1F). The green/red-labeled neurons and their DAPI-stained nuclei were distinguished easily, and their merged result demonstrated the co-location ability of our system with single-cell resolution. These results indicated that our system enabled us to detect two specific fluorescence-labeled neuronal structures and their co-located cytoarchitecture without crosstalk.

### Correction of lateral chromatic aberration

**FIGURE 2:**
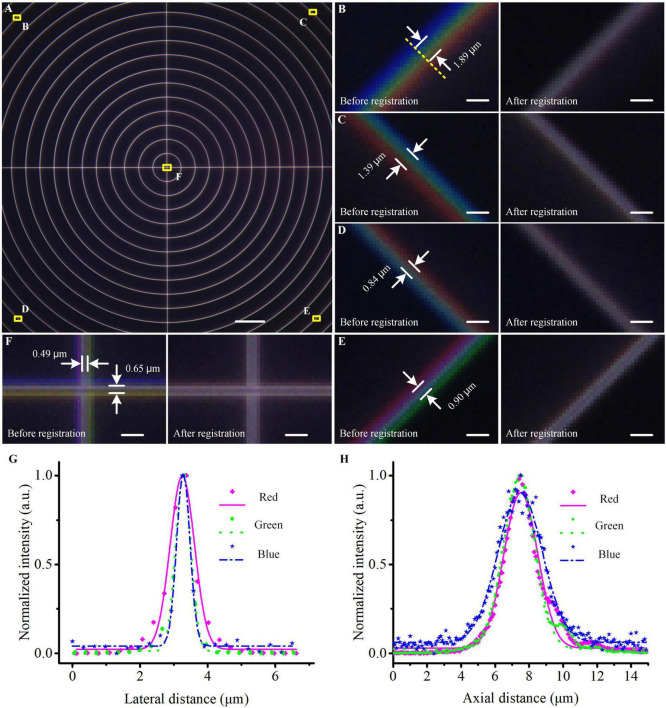
Co-located correction and optical resolution of the multicolor WVT system. **(A)** Merged image of the concentric ring sample acquired in blue, green, and red channels before registration. **(B–F)** Enlarged views of the areas are indicated by the corresponding yellow squares in **(A)**. **(G,H)** Lateral and axial optical resolutions of each channel. The points indicated the measured values, the lines indicated the Gaussian fittings. Scale bars: **(A)** 50 μm, **(B–F)** 2 μm.

To eliminate the lateral chromatic aberration in the system, we imaged a concentric ring sample slide (Argo-HM, Argolight, France) in three channels to figure out their co-located errors among three channels (Figure 2A). Blue, green, and red fluorescent signals were emitted from the same concentric rings and their maximum diameter was larger than the entire FOV (576 × 576 μm^2^). We merged the raw images of three channels at four corners and the center of the FOV before registration (Figures 2B–F), and found that the maximum co-located errors at the corners and center of the FOV were 1.89 μm and 0.65 μm, respectively. According to the measurement results, we fixed the image of the green channel, then shifted, rotated, and scaled the images of the other two channels until the cross-correlation coefficient between fixed and transformed images reached the maximum, and the registration parameters were recorded. Then, for the data lateral registration of the imaged mouse brain or other biological samples, we directly transformed each single-FOV image in blue/red channels using the recorded registration parameters to eliminate co-located errors. After that, these lateral co-located errors were reduced to less than 1 pixel (0.32 μm) in the entire FOV (“After registration” images in Figures 2B–F), guaranteeing a subsequent accurate conjoint data analysis of three channels at submicron-colocation resolution. This experiment would be repeated if any adjustment of the optical configuration before the formal data acquisition.

To evaluate the optical resolution of three channels, we imaged blue/green/red monochrome microspheres (365/415; 505/515; 580/605, Molecular Probe, USA) with 0.2 μm diameter. We imaged the beads in three channels with a pattern period of 109.44 μm on the DMD and a Z step of 200 nm. The SIM-reconstructed images were generated in the green and red channels and the WF-reconstructed image was generated in the blue channel. Then we performed Gaussian fitting on the lateral and axial intensity distributions of the fluorescent beads and measured their full width at half maximum (FWHW) values (Figures 2G,H). The lateral and axial resolutions (*n* = 10, mean ± s.e.m) were 0.48 ± 0.01 μm and 3.08 ± 0.03 μm in the blue channel, 0.53 ± 0.01 μm and 2.22 ± 0.06 μm in the green channel, and 0.84 ± 0.03 μm and 2.42 ± 0.06 μm in the red channel.

### Automatic contour recognition to reduce imaging time

**FIGURE 3:**
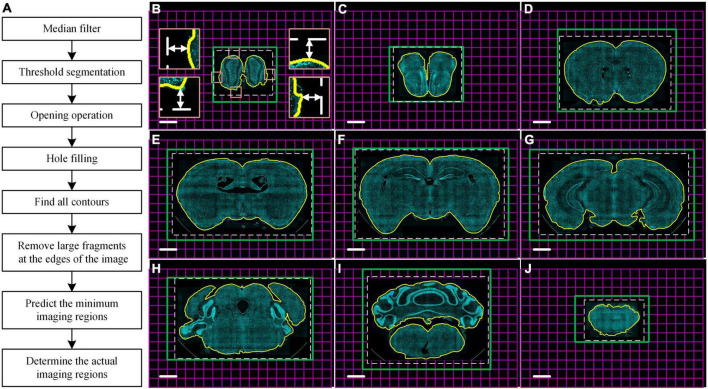
Real-time automatic contour recognition of the whole-brain imaging. **(A)** Flow diagram. **(B–J)** Typical contour recognition results in the whole brain with a 1.25 mm axial interval based on DAPI-staining cytoarchitecture. Yellow curves indicate the identified contour of the brain sample in the raw data. White dotted boxes indicate the minimum imaging regions of the next layer predicted by the real-time automatic contour recognition. Four insets in **(B)** enlarge the corresponding views of the tangency point of the circumscribed rectangle of the sample indicated by pink squares in **(B)**. Double-headed arrows in the four insets of **(B)** indicate a distance of 288 μm from the tangency point to the defined minimum imaging range. Green boxes indicate the actual imaging regions of the corresponding next layers. Each magenta square indicates single FOV. These images are recorded at the same coordinates to ensure the auto-registration of the whole dataset. Scale bars: **(B–J)** 1 mm.

To improve the efficiency of multicolor whole-brain imaging, we developed a program that can obtain a real-time minimum imaging range defined by automatic brain contour recognition using cytoarchitecture information. The imaging area usually is defined as a cuboid covering the maximum cross-section of the whole brain to ensure the integrity of the acquired data due to irregular brain outlines. However, this leads to extra redundant imaging of surrounding embedding agents and then extending of total data acquisition time. Our algorithm of automatic contour recognition was based on mathematical morphology image processing. It was executed by the idle compute resource of the data acquisition workstation during every microtome sectioning. The flow diagram of the algorithm was shown in Figure 3A. Briefly, the latest complete coronal image of DAPI-staining cytoarchitecture as the input image was sequentially subjected to median filtering, threshold segmentation, and opening operations, so that false positive signals from image noise, tissue fragments generated by sectioning, and boundary of embedded agents were removed from the image. Then, the hole-filling operation was performed to fill large hollow areas in the brain, such as the ventricle. After the above processing, the foreground area of the image just covered the brain tissue area, so the boundary of the foreground was regarded as the brain contour (yellow curve in Figures 3B–J). To ensure the data integrity, the minimum rectangular imaging region was predicted by defining its four boundaries 288 μm (half the size of the FOV) away from the circumscribed rectangular of the brain contour (white dotted boxes in Figures 3B–J). Then, the actual imaging region of the next layer was performed by adjusting the corresponding imaging mosaic numbers (green boxes in Figures 3B–J). The algorithm usually took less than 5 s to recognize the brain contour of one layer at a pixel size of 1.68 × 1.68 μm^2^, shorter than the microtome sectioning time of 24 s. Therefore, it enabled the real-time prediction of the imaging range of the next layer. The real-time counterstaining with DAPI achieved a uniform staining effect throughout the whole brain and provided the consistent data quality of each layer. This led to excellent and stable contour recognition in whole-brain optical imaging.

To verify the recognition accuracy of the algorithm, we imaged eight 8-week-old C57BL/6J male mice brains with DAPI counterstaining at a voxel size of 0.32 × 0.32 × 2 μm^3^ and recognized the coronal brain contour of all layers. The results showed that our algorithm could recognize the brain contour at different locations throughout the brain (Figures 3B–J). According to the recognition results, we further compared the number of imaged mosaics with and without contour recognition in the multicolor WVT imaging (Supplementary Table 1). Each mosaic image of a single FOV was reconstructed by three images with different structured illumination patterns. The number of mosaics of each layer without contour recognition was estimated using the maximum coronal plane of the whole brain, and the layer number was the same as the total number of layers acquired with automatic contour recognition. The average mosaic number in each channel was 858,701 and 546,310 without and with automatic contour recognition, respectively. The average proportion of the reduced mosaics number was 37%, and the data acquisition time (not including sectioning time) and storage space were also reduced by 37%. The exposure number of each camera and the flipping times of the DMD fall by 937,173 (3 × mosaic reduction) with automatic contour recognition. This showed that the losses of DMD life and camera life could be significantly reduced when automatic contour recognition was used. The mosaic image size was 6.18 MB with 16-bit image depth, so the average size of a raw data set that contains three channels was 15.2 TB without automatic contour recognition, and 9.7 TB with automatic contour recognition. The imaging time of each mosaic image included the exposure time of the camera, the flipping time of DMD, the movement and stabilization time of the three-dimensional (3D) translation stage and piezoelectric translational stage, image reconstruction time by SIM, and image saving time. When the exposure time of the camera was set to 50 ms, the average imaging time of each mosaic in the multicolor WVT was 342.5 ms. So, the imaging time of the whole brain was 81.7 h without automatic contour recognition, and 52.0 h with automatic contour recognition.

### Identification and quantitative analysis of type-specific and projection-specific neurons and their relationship at the whole-brain level

Dissecting the distribution characteristics of neural circuits and identifying the neuronal types is vital for understanding brain function. To demonstrate the application prospects of the multicolor WVT in this regard, we acquired the brain-wide distribution of cholinergic neurons and the inputs of the ventral CP with co-located cytoarchitecture in the same brain.

**FIGURE 4:**
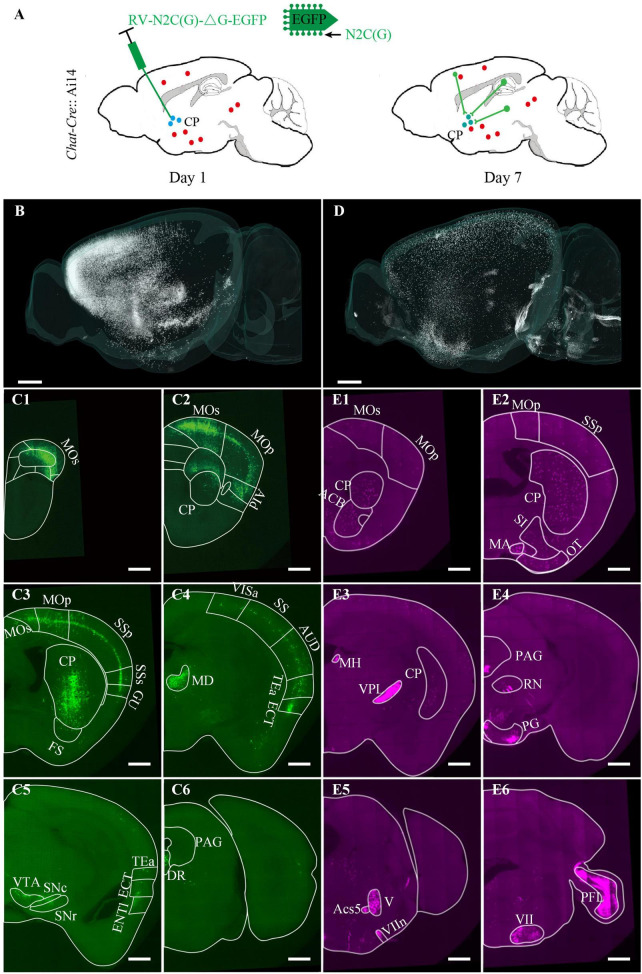
Brain-wide distribution of the cholinergic neurons and the inputs of the ventral CP. **(A)** Labeling schematic of the inputs of the ventral CP in *Chat-Cre*: Ai14 transgenic mouse. **(B)** Brain-wide signal distribution in the green channel. **(C1–C6)** Representative signal distribution in the green channel at typical coronal planes. **(D)** Brain-wide signal distribution in the red channel. **(E1–E6)** Representative signal distribution in the red channel at typical coronal planes. Typical brain regions of interest were marked. The projection thickness of the images: **(C1–C6)** 10 μm, **(E1–E6)** 40 μm. Scale bars: **(B,D)** 1 mm, and **(C1–C6,E1–E6)** 500 μm.

**FIGURE 5:**
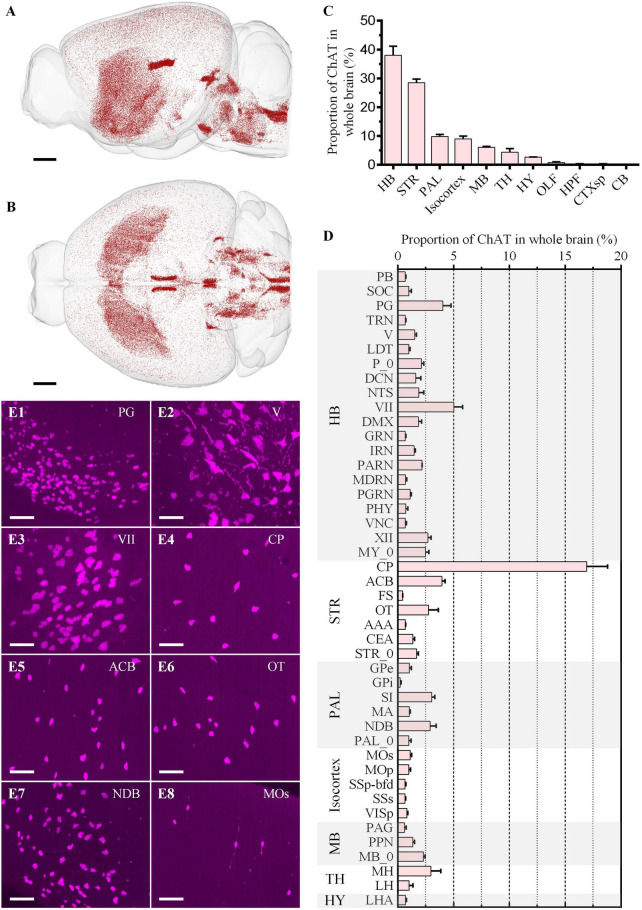
Whole-brain quantitative statistics of the cholinergic neurons in the red channel. **(A,B)** Whole-brain distribution of cholinergic neurons in the sagittal view **(A)** and horizontal view **(B)**. **(C,D)** The proportion of the cholinergic neurons in brain regions **(C)** and brain sub-regions **(D)**. Data is shown as mean ± s.e.m (*n* = 4 mice). **(E1–E8)** Signal distribution in PG, V, VII, CP, ACB, OT, NDB, and Mos, respectively. The projection thickness of the images: **(E1–E8)** 50 μm. Scale bars: **(A,B)** 1 mm, and **(E1–E8)** 50 μm.

The virus-labeling principle was shown in Figure 4A. Briefly, the *Chat-Cre* transgenetic mice were crossed with the Ai14 reporter mice to label the cholinergic neurons with tdTomato (*Chat-Cre*: Ai14). Then, 100 nl N2C (G) coated rabies virus expressing EGFP was injected into the ventral CP to label the input neurons. One week later after the virus injection, all mice were perfused with PBS and PFA. The brains were removed from the skull and embedded in resin for subsequent imaging. We imaged the brains at a voxel size of 0.32 × 0.32 × 2 μm^3^ for about 3 days. We acquired the DAPI-staining cytoarchitecture in the blue channel, the input distribution of the ventral CP in the green channel (Figures 4B, C1–C6), and the distribution of cholinergic neurons in the red channel (Figures 4D, E1–E6). Overall, the input signal was mainly distributed in the Isocortex and striatum of the anterior brain (Figure 4B). In the posterior brain, there was a dense input signal in MD (Full name of brain areas were shown in Supplementary Table 2) and sparse input signals in VTA, SNc, SNr, and DR (Figures 4C4–C6). In contrast to the input distribution of CP, the distribution of cholinergic neurons was mainly in the striatum and pallidum (Figure 5D). The brain regions with dense soma signals included MA, MH, PAG, PG, V, Ecs5, VII, etc., and with sparse soma signals included Isocortex, CP, ACB, SI, OT, etc. (Figures 4E1–E6). The dense neural fiber signal was mainly distributed in VPL, VIIn, and PFL (Figures 4E5,E6).

**FIGURE 6:**
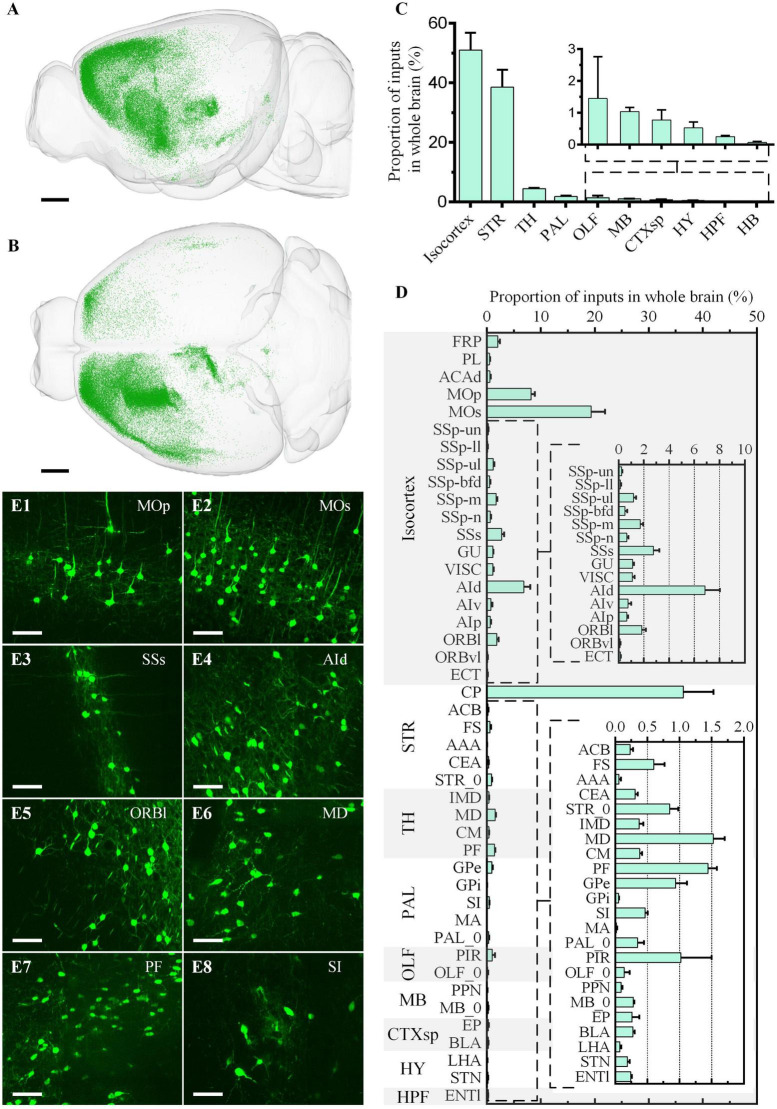
Whole-brain quantitative statistics of the inputs of the ventral CP in the green channel. **(A,B)** Whole-brain distribution of input neurons in the sagittal view **(A)** and horizontal view **(B)**. **(C,D)** The proportion of inputs in the brain regions **(C)** and the brain sub-regions **(D)**. Data is shown as mean ± s.e.m (*n* = 4 mice). **(E1–E8)** Signal distribution in MOp, MOs, SSs, AId, ORBl, MD, PF, and SI, respectively. The projection thickness of the images: **(E1–E8)** 10 μm. Scale bars: **(A,B)** 1 mm, and **(E1–E8)** 50 μm.

We then identified all green (Figures 6A,B) and red (Figures 5A,B) neurons in the whole brain and registered them to the Allen Common Coordinate Framework (CCFv3) based on DAPI-staining cytoarchitecture (Supplementary Figure 3). The proportion of the input neurons of CP and cholinergic neurons in each brain region in the whole brain was quantified automatically (Figures 5C,D, 6C,D).

For the input neurons, the highest proportion of neurons was 50.98% ± 5.81% in Isocortex, the second-highest proportion was 38.57% ± 5.79% in STR, and the proportion of the other regions was less than 5.00% (Figure 6C). In Isocortex, the input neurons mainly came from MOp (Figure 6E1), MOs (Figure 6E2), and AId (Figure 6E4), whose proportions all were more than 5.00%. In STR, the proportion of input neurons in CP, which also represent local input, was 36.39% ± 5.57%. The input neurons proportion of other brain regions in STR was far less than in CP, less than 1.00%. In TH, the two brain regions with the highest proportion were MD (Figure 6E6) and PF (Figure 6E7), 1.52% ± 0.18% and 1.45% ± 0.14%, respectively. In PAL, the input neurons were mainly distributed in GPe and SI (Figure 6E8), whose proportions were 0.94% ± 0.17% and 0.47 ± 0.04%, respectively. In OLF, the input neurons were mainly distributed in PIR, whose proportion was 1.02% ± 0.48%.

The distribution of cholinergic neurons was symmetrical between the left and right hemispheres (Figure 5B). In general, neuron density could be divided into three grades: MH, LDT, and VII, etc. with the highest density, STR and PAL, etc., with the moderate density, and Isocortex with the lowest density (Figures 5A,B). Most cholinergic neurons were distributed in HB, STR, PAL, Isocortex and MB, whose proportion were 37.96% ± 3.16%, 28.51% ± 1.24%, 9.80% ± 0.78%, 9.01% ± 0.95% and 6.09% ± 0.30%, respectively (Figure 5C). The proportion of the other brain regions was all less than 5.00%. Specifically, only in CP, its cholinergic neurons account for more than 5.00% of the total cholinergic neurons in the whole brain as a brain sub-region (Figure 5D). The brain sub-regions with a proportion between 2.50% and 5.00% included PG, VII, XII, ACB, OT, SI, NDB, and MH. Typical signal distributions were shown in Figures 5E1–E8. Though the number of cholinergic neurons in CP was the largest, its cholinergic neuron density was relatively low (Figure 5E4). In contrast, the density in PG (Figure 5E1), V (Figure 5E2), VII (Figure 5E3), and NDB (Figure 5E7) were higher.

**FIGURE 7:**
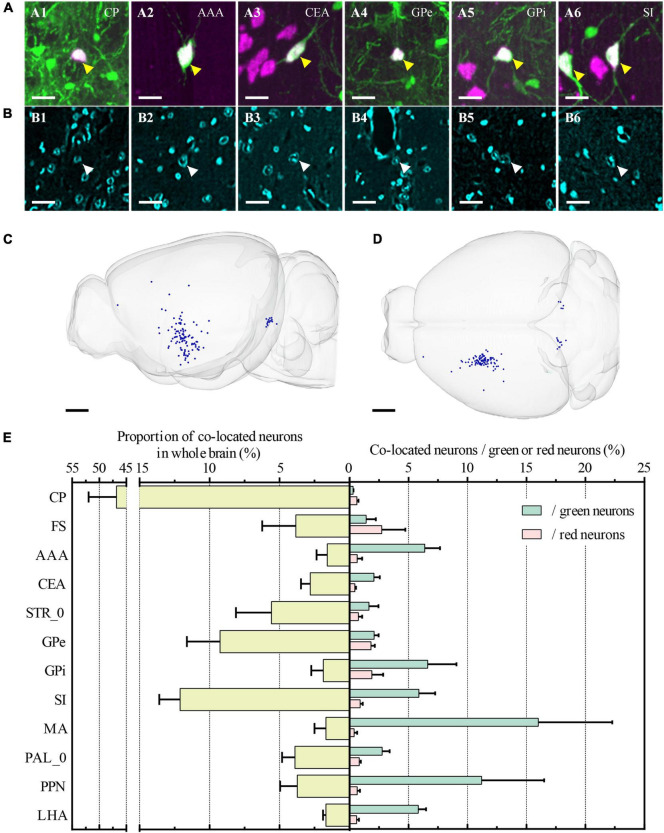
Whole-brain quantitative statistics of co-located neurons between green and red channels. **(A1–A6)** Merged images of the co-located neurons at CP, AAA, CEA, GPe, GPi, and SI, respectively. Green and magenta represent signals in the green and red channels, respectively. The yellow arrows indicated the co-located neurons. **(B1–B6)** Images were acquired in the blue channel at the same region of interest in **(A1–A5)**. The white arrows indicated the nucleus of co-located neurons that were indicated in **(A1–A5)**. The projection thickness of the images: **(A1–A6)** 40 μm and **(B1–B6)** 2 μm. **(C,D)** Whole-brain distribution of co-located neurons in sagittal view **(C)** and horizontal view **(D)**. **(E)** The yellow histogram on the left showed the proportion of the co-located neurons in the main brain sub-regions, and the green and red histogram on the right showed the proportion of co-located neurons to the input neurons and the cholinergic neurons, respectively. Data is shown as mean ± s.e.m (*n* = 4 mice). Scale bars: **(A1–A6, B1–B6)** 20 μm and **(C,D)** 1 mm.

Furthermore, we also identified all co-located neurons among the three channels (Figures 7A1–A6, B1–B6). The co-located neurons between the green and red channels represented that the type of input neurons of CP was cholinergic. Therefore, the green input neurons without a red co-located signal indicated the non-cholinergic input neurons, while the red cholinergic neurons without a green co-located signal represented the cholinergic neurons that didn’t project to ventral CP. Overall, the number of co-located cholinergic input neurons of CP was relatively small in the whole brain (Figures 7C,D). The proportion of co-located neurons to green input neurons and red cholinergic neurons were 0.22% ± 0.04% and 0.22% ± 0.05%, respectively. Co-located cholinergic input neurons of CP were mainly distributed in STR, PAL, MB, and HY (The left histogram in Figure 7E). The top-five brain sub-regions for the proportions of co-located neurons were CP with 46.84% ± 5.13%, SI with 12.11% ± 1.49%, GPe with 9.25% ± 2.37%, STR_0 with 5.58% ± 2.54%, and PAL_0 with 3.90% ± 0.93%. The proportion of co-located neurons to green input neurons is shown as a green histogram on the right of Figure 7E. The top-five brain regions were MA with 16.00% ± 6.29%, PPN with 11.20% ± 5.31%, GPi with 6.62% ± 2.44%, AAA with 6.37% ± 1.30%, and SI with 5.88% ± 1.37%. The proportion of co-located neurons to red cholinergic neurons is shown as a red histogram on the right of Figure 7E. The top-five brain regions were FS with 2.75% ± 2.00%, GPi with 1.89% ± 0.96%, GPe with 1.82% ± 0.32%, SI with 0.89% ± 0.22%, and PAL_0 with 0.81% ± 0.15%.

**FIGURE 8:**
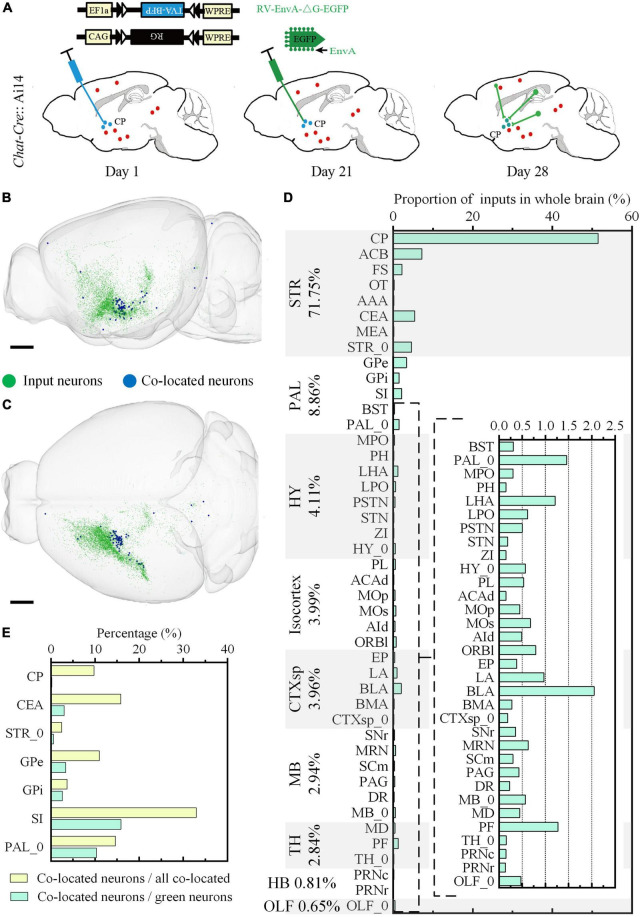
Whole-brain quantitative statistics of the inputs of the cholinergic neurons in the ventral CP. **(A)** Labeling schematic of the inputs of the cholinergic neurons in the ventral CP. **(B,C)** Whole-brain distribution of the input neurons and co-located neurons in sagittal view **(B)** and horizontal view **(C)**. **(D)** The proportion of the inputs in typical brain sub-regions. The percentage to the left of the image indicated the proportion of the inputs in typical brain regions. **(E)** The yellow histogram represented the proportion of the co-located neurons in brain sub-regions, and the green histogram represented the proportion of the co-located neurons to the inputs of the cholinergic neurons in the ventral CP. Scale bars: **(B,C)** 1 mm.

We further studied the whole-brain input distribution of cholinergic neurons in ventral CP. The labeling principle was shown in Figure 8A. Briefly, 150 nl Cre dependent AAV helper virus expressing histone tagged BFP and TVA, as well as the RG (AAV-DIO-hisBFP-TVA and AAV-DIO-RG mixture, 1:2 at volume rate), was injected into the ventral CP of the *Chat-Cre*: Ai14 mouse. Three weeks after the virus injection, 200 nl EnvA coated rabies virus expressing EGFP was injected into the same site to label the long-range input neurons of the cholinergic neurons. The input distribution of cholinergic neurons in the ventral CP was acquired in the green channel, and the types of signals in the other two channels were the same as the previous one. Unlike the input distribution of Figures 4, 6 Isocortex was no longer dominant in the inputs of cholinergic neurons in ventral CP (Figures 8B–D). The top-five proportions of the input neurons were 71.75% in STR, 8.86% in PAL, 4.11% in HY, 3.99% in Isocortex, and 3.96% in CTXsp. The brain sub-regions with a high proportion were mainly in STR and PAL (Figure 8D), also different from the result in Figure 6. The proportion in CP of input neurons to the total input neurons of cholinergic neurons in ventral CP was 51.48%. It indicated that cholinergic neurons in ventral CP mainly received local input connections. Other brain sub-regions with a proportion over 2.50% included ACB (7.23%), CEA (5.42%), STR_0 (4.59%), and GPe (3.37%). The distribution of the co-located neurons was sparse, and their positions (Figures 8B,C) were consistent with the result in Figures 7C,D. Overall, the proportion of co-located neurons to the total input neurons of cholinergic neurons in ventral CP was 1.03%, also higher than the result in Figure 4. The co-located neurons were mainly in STR and PAL, the top-five proportions were 32.93% in SI, 15.85% in CEA, 14.63% in PAL_0, 10.98% in GPe, and 9.76% in CP (Figure 8E). The top-five proportions of co-located neurons to the green input neurons were 15.88% in SI, 10.34% in PAL_0, 3.36% in GPe, 3.02% in CEA, and 2.59% in GPi (Figure 8E).

**FIGURE 9:**
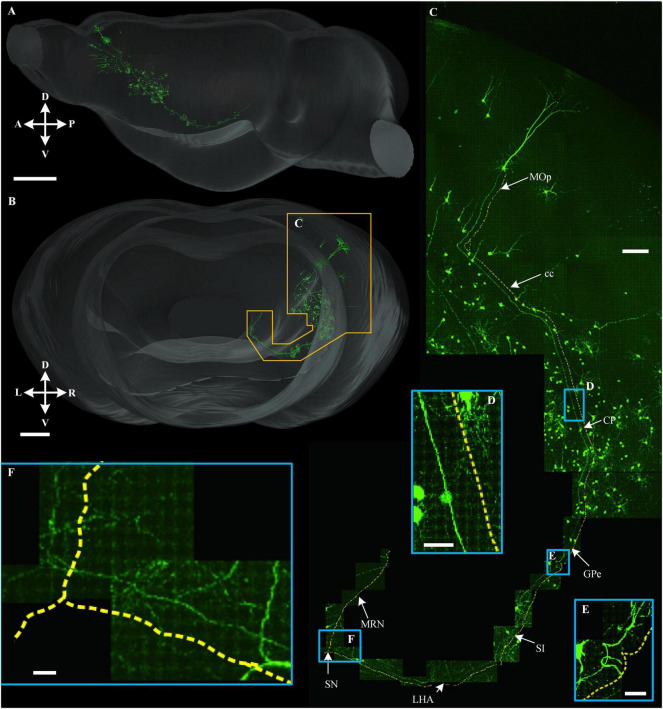
Long-range tracing of a neuron that projected to the cholinergic neuron in ventral CP. **(A,B)** 3D reconstruction of a long-range projection neuron to the cholinergic neuron in ventral CP and the signal distribution near this neuron in sagittal view **(A)** and horizontal view **(B)**, respectively. **(C)** An enlarged view of the area is indicated by the orange special-shaped box in **(B)**. The yellow dotted line was parallel with a long-range fiber path from the soma. **(D–F)** Enlarged views of the area are indicated by the corresponding blue boxes in **(C)**. Scale bars: **(A)** 1 mm, **(B)** 500 μm, **(C)** 100 μm, and **(D–F)** 20 μm.

To demonstrate the continuity of the long-range projection fibers acquired in the multicolor WVT, we traced a long-range projection pattern of the neuron in MOp projecting to the cholinergic neurons in the ventral CP (Figure 9). The projection range was 1.1 mm along the left to right (LR) direction, 2.5 mm along the dorsal to ventral (DV) direction, 3.9 mm along the anterior and posterior (AP) direction, and the total length including dendrites and axons was 15.2 mm. We then aligned this neuron in the whole brain and found its axon successively passed through cc, CP, GPe, SI, LHA, SN, and MRN. This indicated that cholinergic neurons in the ventral CP play a role in motor control.

## Discussion

Here, we reported a multicolor high-resolution high-throughput whole-brain imaging method to acquire two kinds of fluorescence-labeled neural structures in green/red channels and co-located DAPI-staining cytoarchitecture in the blue channel simultaneously.

Upgrading whole-brain optical imaging to multicolor is not simply a matter of increasing the number of channels. In practice, it encounters challenges in technical details. Considering the terabytes magnitude of data acquisition per sample, the desire for imaging throughput requires that multiple channels must be imaged simultaneously. However, the axial chromatic aberration and spectra mixing between different channels affect the realization of multicolor high-resolution whole-brain optical imaging. Based on analyzing the imaging characteristics and image quality of different channels, we designed a multicolor whole-brain imaging scheme to overcome these two technical bottlenecks by smartly utilizing axial chromatic aberration and different imaging modes. This optimized scheme does not require further hardware modification of the existing multicolor WVT system and avoids increasing the complexity of the system.

In recent years, whole-brain optical imaging technology has also developed rapidly. ChroMS achieved 3D multicolor imaging with the integration of tri-chromatic two-photon excitation by wavelength mixing into an STP system (Mahou et al., 2012). However, its imaging throughput was limited by point-scanning mode, and therefore, they only showed the 3D imaging results of over several cubic millimeters as well as brain-wide serial 2D multichannel imaging with an axial step of 100 μm. Our group also reported a chemical sectioning imaging principle and presented a whole-brain imaging result of the dual-fluorescence-labeled sample (Wang et al., 2021). To our knowledge, acquiring and quantifying two kinds of interesting information and their relationship with the anatomical annotation in the same whole brain hasn’t been demonstrated by high-resolution whole-brain optical imaging technologies. Our work filled this technical gap and demonstrated the application potential by deciphering the relationship between the upstream and downstream neural circuits in the same brain.

Striatal cholinergic neurons comprise a small population of cells but have fundamental roles in fine-tuning basal ganglia functions, which are essential in synaptic plasticity and motor learning. But how the striatum processes cortical inputs are not very clear. Using multicolor WVT imaging, we compared the input of striatal cholinergic neurons and non-cholinergic neurons. Firstly, we quantitatively analyzed the brain-wide distribution of cholinergic neurons and the input pattern of the ventral CP in the same brain. Then, we identified the type of upstream neurons that projected to the ventral CP through co-location green/red channels without immunostaining. Further, we acquired the input pattern of cholinergic neurons in the ventral CP and found that it and the input patterns of all types of neurons were different in Isocortex. It implied that many neurons from the Isocortex project to the non-cholinergic neurons in ventral CP. In addition, we found the proportion of co-located cholinergic neurons to input neurons of cholinergic neurons in CP was 4.7 times the proportion of co-located cholinergic neurons to input neurons of CP. It implied that the connection from long-range cholinergic neurons to cholinergic neurons was stronger than the one to the non-cholinergic neurons in the ventral CP.

In summary, we developed the multicolor WVT to obtain high-resolution whole-brain fluorescence images of different interesting targets with co-located cytoarchitecture in the same brain. Combined with advanced transgenetic and virus labeling technologies, our multicolor WVT system is potential to map the fine structures of different components in the complex neural network and decipher their inner relationships. For example, combined with *c-fos* transgenetic mice and the RV tracing method, we might map the activity-dependent neural network in the whole brain. Acquiring the whole morphology of the inputs and output neurons in the specific regions in the same brain might draw the complete neural circuit diagram of specific information flow. All these attempts will promote the understanding of how the brain dynamically organizes multi-region to achieve a specific function.

## Materials and methods

### Animals

An 8-week-old *Chat-Cre*: Ai14 transgenic male mouse and an 8-week-old C57BL/6J male mouse (Jackson Laboratory, Bar Harbor, ME, USA) were used in section “Optimization of multicolor imaging scheme for overcoming axial chromatic aberration and spectral mixing,” eight 8-week-old C57BL/6J male mice were used in section “Automatic contour recognition to reduce imaging time,” five *Chat-Cre*: Ai14 transgenic male mice were used in section “Identification and quantitative analysis of type-specific and projection-specific neurons and their relationship at the whole-brain level.” The *Chat-Cre*: Ai14 transgenic male mouse was generated by crossing the *Chat-Cre* transgenetic mouse (Jackson Laboratory, Bar Harbor, ME, USA) with the Ai14 reporter mouse (Jackson Laboratory, Bar Harbor, ME, USA).

All mice used in this study were housed in normal cages in an environment with a 12-h light/dark cycle with food and water *ad libitum*. All animal experiments were approved by the Animal Ethics Committee of the Huazhong University of Science and Technology.

### Virus injection

Five types of the virus were used and purchased from Wuhan BrainVTA Co., Ltd., China. They were RV-N2C(G)-ΔG-EGFP (5 × 10^8^ IFU/mL), RV-EnvA-ΔG-EGFP (5 × 10^8^ IU/ml), rAAv-EF1α-DIO-mCherry-TVA (2∼2.5 × 10^12^ vg/ml), rAAv-EF1α-DIO-BFP-TVA (2∼2.5 × 10^12^ vg/ml), and rAAV- EF1α-DIO-oRG (2∼2.5 × 10^12^ vg/ml).

All mice were deeply anesthetized by intraperitoneally injected (100 g/ml) with 2% chloral hydrate and 10% urethane-configured anesthetic before they were mounted and microinjected with a stereotaxic system.

For dual-color labeling in section “Optimization of multicolor imaging scheme for overcoming axial chromatic aberration and spectral mixing,” 150 nl of Cre-dependent AAV helper virus expressing mCherry and TVA, as well as the RG (rAAv-EF1α-DIO-mCherry-TVA and rAAV- EF1α-DIO-oRG mixture, 1:2 at volume rate), was injected into CP (AP 0.145 mm, ML 2.0 mm, DV –4.85 mm) of an 8-week-old *Chat-Cre*: Ai14 mouse. Three weeks after the virus injection, 200 nl EnvA coated rabies virus expressing EGFP (RV-EnvA-ΔG-EGFP) was injected into the same site.

For labeling the inputs of the ventral CP (*n* = 4 mice) in section “Identification and quantitative analysis of type-specific and projection-specific neurons and their relationship at the whole-brain level,” 100 nl of RV-N2C(G)-ΔG-EGFP was injected into the ventral CP (AP 0.145 mm, ML 2.0 mm, DV –4.85 mm) of the 8-week-old *Chat-Cre*: Ai14 mice. For labeling the inputs of the cholinergic neurons in ventral CP (n = 1 mouse) in section “Identification and quantitative analysis of type-specific and projection-specific neurons and their relationship at the whole-brain level,” 150 nl Cre dependent AAV helper virus expressing histone tagged BFP and TVA, as well as the RG (rAAv-EF1α-DIO-BFP-TVA and rAAV- EF1α-DIO-oRG mixture, 1:2 at volume rate), was injected into the ventral CP (AP 0.145 mm, ML 2.0 mm, DV –4.85 mm) of an 8-week-old *Chat-Cre*: Ai14 mouse. Three weeks after the virus injection, 200 nl EnvA coated rabies virus expressing EGFP (RV-EnvA-ΔG-EGFP) was injected into the same site.

After the surgery, the incisions were stitched and lincomycin hydrochloride and lidocaine hydrochloride gel were applied to prevent inflammation and alleviate pain for the animals. One week after virus injecting, all mice were perfused with PBS and PFA.

### Tissue preparation

All histological procedures have been previously described (Gong et al., 2013; Yang et al., 2013; Peng et al., 2017). Briefly, the mouse was deeply anesthetized using a 1% solution of sodium pentobarbital and subsequently intracardially perfused with 0.01M PBS (Sigma-Aldrich Inc., USA), followed by 4% paraformaldehyde (Sigma-Aldrich Inc., USA) in 0.01M PBS. The brains were post-fixed in 4% paraformaldehyde at 4^°^C for 24 h. After fixation, each intact brain was rinsed overnight at 4°C in a 0.01M PBS solution and subsequently dehydrated in a graded ethanol series (50, 70, and 95% ethanol, changing from one concentration to the next every 1 h at 4^°^C). After dehydration, the brains were immersed in a graded glycol methacrylate (GMA) series (Ted Pella Inc., Redding, CA, USA), including 0.2% SBB (70, 85, and 100% GMA for 2 h each and 100% GMA overnight at 4^°^C). Subsequently, the samples were impregnated in a pre-polymerization GMA solution for 3 days at 4^°^C and embedded in a vacuum oven at 48^°^C for 24 h. The 100% GMA solution comprised 67 g of A solution, 2.8 g of deionized water, 29.4 g of B solution, 0.2 g of SBB, and 0.6 g of AIBN as an initiator. The 70 and 85% GMA solutions (wt⋅wt^–1^) were prepared from 95% ethanol and 100% GMA.

### Instrument

The multicolor WVT is shown in Figure 1A, upgraded from an original dual-color WVT system (Gong et al., 2016). Light generated from a mercury lamp (X-cite Exacte, Lumen Dynamics, Canada) was guided by a liquid light guide, then was collimated and oriented by a lens (f = 40 mm, AC254-040-A, Thorlabs, USA) toward the DMD (XD-ED01N, X-digit, China). The DMD was a 1,024 × 768 micromirror array with the 13.68 × 13.68 μm^2^ single micromirror. It was used to display three binary periodic fringe patterns with different phases successively. The micromirror of the DMD was conjugate with the focal plane of the objective *via* a tube lens (f = 150 mm, AC254-150-A-ML, Thorlabs, USA) and a 20 × water-immersion objective (1.0 NA, XLUMPLFLN 20XW, Olympus, Japan). The filter set (DA/FI/TR-A, Semrock, USA) near the objective realized 3-band filtering of excitation light and emission light. Fluorescence emitted from the sample was imaged by objective and tube lens (U-TLU, Olympus, Japan. TL2 in Figure 1A), then separated by two dichroic mirrors (DM, FF484-FDi01 and FF560-FDi01, Semrock, USA) and further filtered by three filters (FF02-435/40, FF01-523/20, FF01-593/LP Semrock, USA). Three scientific complementary metal-oxide-semiconductor cameras (ORCA-Flash 4.0, Hamamatsu Photonics K.K., Japan) were used to acquire the signals in three channels simultaneously. The sensor array of the camera was 2,048 × 2,048 pixels with a 6.5 μm pixel size. Axial scanning was achieved by a piezoelectric translational stage (P-725.1CD, Physik Instrumente, Germany). The actual imaging format was set to 1,800 × 1,800 pixels to fit the size of the modulated light field of the DMD. The sample box was screwed onto a high-precision 3D translation stage (ABL20020-ANT130-AVL125, Aerotech Inc., USA). The 3D translation stage moved the sample for mosaic scanning and sectioning. A 45^°^ diamond knife (Diatome AG, Nidau, Switzerland) was used as a microtome to section the sample. The DMD, piezoelectric translational stage, 3D stage, and cameras were controlled by customized C + + software in a workstation to achieve data acquisition. The acquisition and motion control software was communicated *via* a TCP/IP protocol. The acquired data were saved as TIFF files in a storage array (PowerVault MD1200 with a PERC H810 Host-RAID adapter, Dell Inc., USA).

### Multicolor whole-brain imaging with real-time diamidino-2-phenylindole staining

Whole-brain imaging was performed in the water bath with real-time staining (Gong et al., 2016). The sample was immersed in a water bath containing 1 μg⋅ml^–1^ DAPI to achieve whole-brain counterstaining. After sectioning, the dye immediately penetrated the fresh surface, combined with nucleic acid inside the neuron nucleus, and stained the neuron nucleus. We added a little bit of highly concentrated solution of DAPI (50–100 μl solution with a concentration of 1 mg⋅ml^–1^) to balance the concentration of DAPI in the water bath every 6 h during data acquisition.

To determine the position of each detection plane in Figure 1E, we imaged the concentric ring sample slide (Argo-HM, Argolight, France). We adjusted the axial position of the sample by the 3D translation stage until the images of green and red channels were the clearest. Next, we lifted the sample 4 μm and recorded the image of the blue channel. We adjusted the camera of the blue channel until the image was the clearest. Then all detection planes were set well for multicolor imaging.

For formal data acquisition, we adjusted the sample axially until the live images acquired in the blue channel were the clearest, which meant the BDP coincided with the SS and the GDP/RDP was 4 μm below the SS. After these preparations, the imaging parameters were set, and the multicolor WVT automatically performed the sectioning and imaging to complete the brain-wide data acquisition.

For each FOV, three phase-shifted raw images with a phase step of π/2 were acquired in each channel. We reconstructed SIM images with optical sectioning in the green and red channels and WF images by superposing three raw images in the blue channel. When necessary, axial scanning was subsequently executed using the piezoelectric translational stage. Subsequently, the sample was moved to the next mosaic FOV with a 5 μm overlap between the adjacent FOVs. The mosaic imaging process was repeated until the entire coronal section had been acquired. In addition, we used a recirculating filtration device to filter out sections that fell out of the sample and keep the solution pure.

The voxel size of the system was 0.32 × 0.32 × 2 μm^3^. We acquired a z-stack of two images in each FOV after axially scanning at a step of 2 μm and subsequently sectioning at a 4 μm thickness. We achieved the data acquisition of a multicolor whole-brain data set in about 70 h.

### Automatic contour recognition

The principle flow chart is shown in Figure 3A. Firstly, median filtering was done to remove noise from the image. Next, the foreground and background were preliminarily separated by threshold segmentation. The foreground region contained not only the brain tissue sample but also the false positive signals of tissue fragments generated by sectioning and the boundary of the embedded agents. Then, the opening operation was executed to remove these false positive signals in the images, including two steps of corrosion and expansion operation. The algorithm subsequently performed a hole-filling operation to eliminate the interference from large hollow areas in the brain, such as the ventricle. In general, the contours of the brain could be isolated through these operations. However, there were still two challenges. In a few cases, the contours of large fragments around the brain sample were also recognized and interfered with the definition of the imaging range. To improve the robustness of the algorithm, we added an operation based on the distance to eliminate these large fragments far away from the image center. In the other cases, there were still identified boundaries that are slightly smaller than the actual boundaries (Figures 3B–J). To ensure data integrity, the four boundaries of the minimum rectangular imaging regions were set 288 μm (half the size of the FOV) away from the circumscribed rectangular of the brain contour (Four insets in Figure 3B). Benefiting from these above operations, the minimum rectangular imaging regions always completely covered the actual sample regions (white dotted boxes in both Figures 3B–J and Supplementary Figure 4). The actual imaging region of the next layer was then adjusted according to the result of rounding up by adding or subtracting the corresponding mosaics around the four boundaries of the current imaging region (green boxes in Figures 3B–J and brown regions in Supplementary Figure 4). Mosaics-scanning imaging of each layer with the same coordinates ensured the auto-registration of the whole dataset, so we can easily align all the coronal planes with different size imaging regions.

### Cellular localization

NeuroGPS (Quan et al., 2013) software was used to automatically locate soma in the green and red channels in the whole-brain dataset. Since the entire dataset was oversized for the memory capability of a graphics workstation (T7600 with two Intel E5–2687w CPUs, 256GB memory, and an NVIDIA K6000 graphics card, Dell Inc., USA), the dataset was divided into blocks of 512 × 512 × 512 pixels with a voxel size of 1 × 1 × 2 μm^3^. Subsequently, we performed the automatic neuron localization on each block and saved 3D coordinate values in the SWC files. We identified co-localized somas by calculating the distance between the somas in the green and red channels based on the SWC files. If the distance was less than 1 μm, it was identified as a co-located soma. The 3D coordinate values of co-localized neurons were also saved in SWC files. We manually checked all somas and co-located somas after automatic soma localization.

### Image registration and neuron counting

Briefly, 16 brain regions were manually segmented as landmarks based on DAPI-staining raw data with a voxel size of 10 × 10 × 10 μm^3^. Then, BrainsMapi (Ni et al., 2020) software was used to acquire the deformation field for mapping the dataset to the Allen CCFv3 brain atlas. 3D warping of coordinate values of the neurons in SWC files was performed to relocate the anatomical coordinates in the Allen CCFv3. Finally, the number of somas in each brain region was automatically counted.

### Reconstruction of neurons

The neuron in Figure 9 was traced and reconstructed using a semiautomatic approach by GTree (Zhou et al., 2020) software with a voxel size of 1 × 1 × 2 μm^3^. We performed a data format transformation from TIFF to the native TDat format (Li et al., 2017). We started the tracing from the neuron soma, then traversed all the 3D data blocks consecutively along the extension direction of the neurites until all the branches were traced.

## Data availability statement

The raw data supporting the conclusions of this article will be made available by the authors, without undue reservation.

## Ethics statement

The animal study was reviewed and approved by Animal Ethics Committee of the Huazhong University of Science and Technology.

## Author contributions

JY and HG directed the project. JY and ZD designed, constructed the imaging system, and wrote and modified the manuscript. JZ, TL, and JC performed data acquisition. BL and XZ completed the boundary contour extraction. XL, SC, and QS labeled and processed the samples. AL, XJ, JZ, WC, and ZD performed data analysis. All authors contributed to the article and approved the submitted version.
