# Supplementary material for: Multicolor high-resolution whole-brain imaging for acquiring and comparing the brain-wide distributions of type-specific and projection-specific neurons with anatomical annotation in the same brain
Source: Front Neurosci. 2022 Oct 6;16:1033880. doi: 10.3389/fnins.2022.1033880 (PMC9583816; doi:10.3389/fnins.2022.1033880)
Supplement: Supplementary file 1 [file Data_Sheet_1.docx]

Supplementary Material

# Supplementary Figures


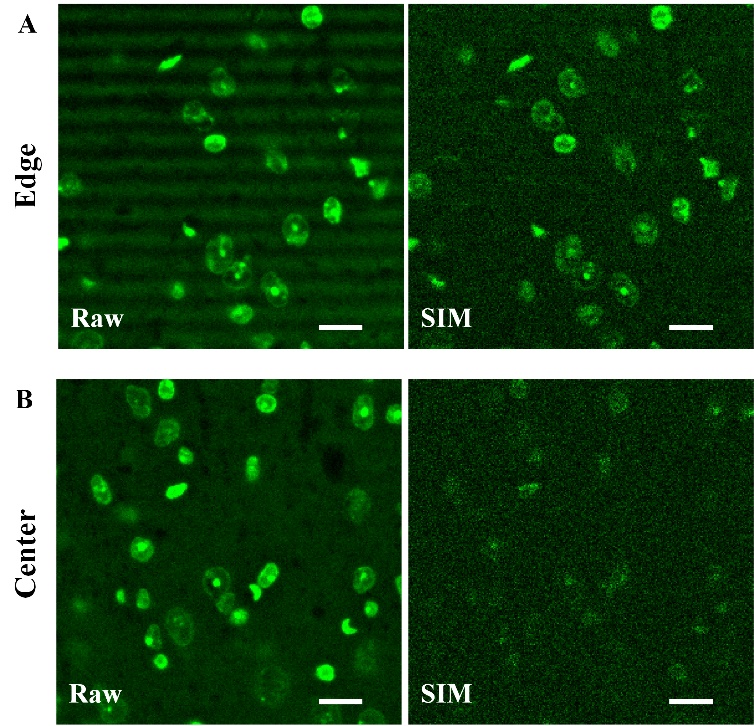


**Supplementary Figure 1.** DAPI crosstalk signals in the green channel at the edge and center of the FOV in “Imaging B” of **Figure 1B**. “Raw” images represented the DAPI raw signals modulated by structured illumination, and “SIM” images represented the SIM-reconstructed optical-sectioning images. Scale bars: 10 μm.


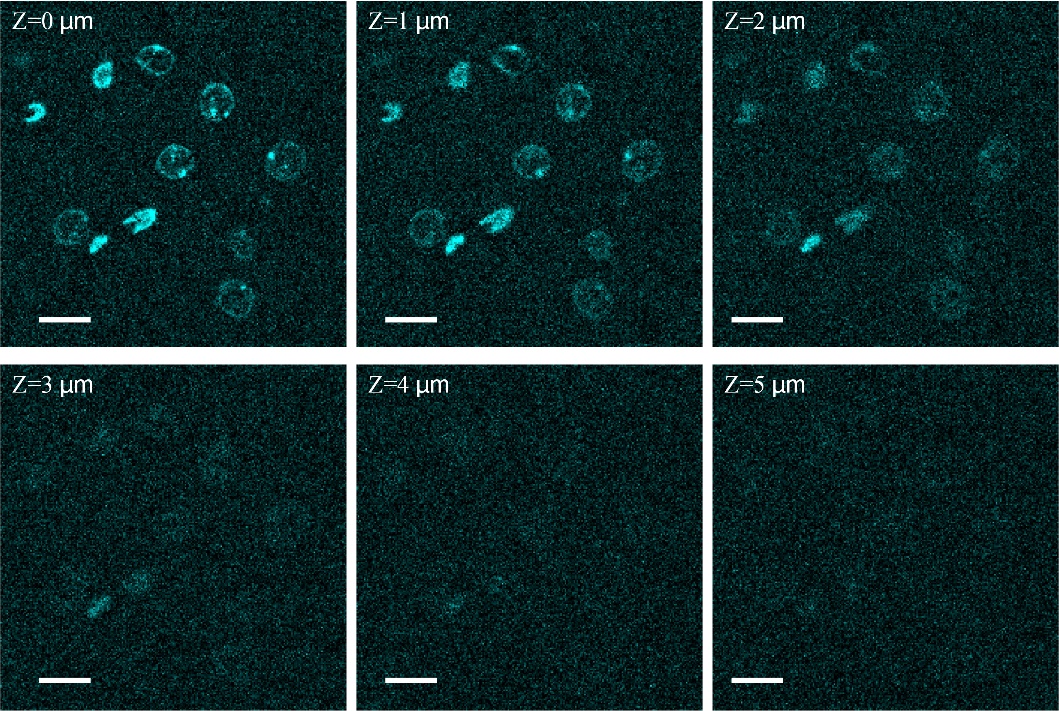


**Supplementary Figure 2.** DAPI staining signals at different imaging depths in the blue channel. The concentration of DAPI solution was 1 μg∙ml^-1^, and the staining time was 30 s. Scale bars were 10 μm.


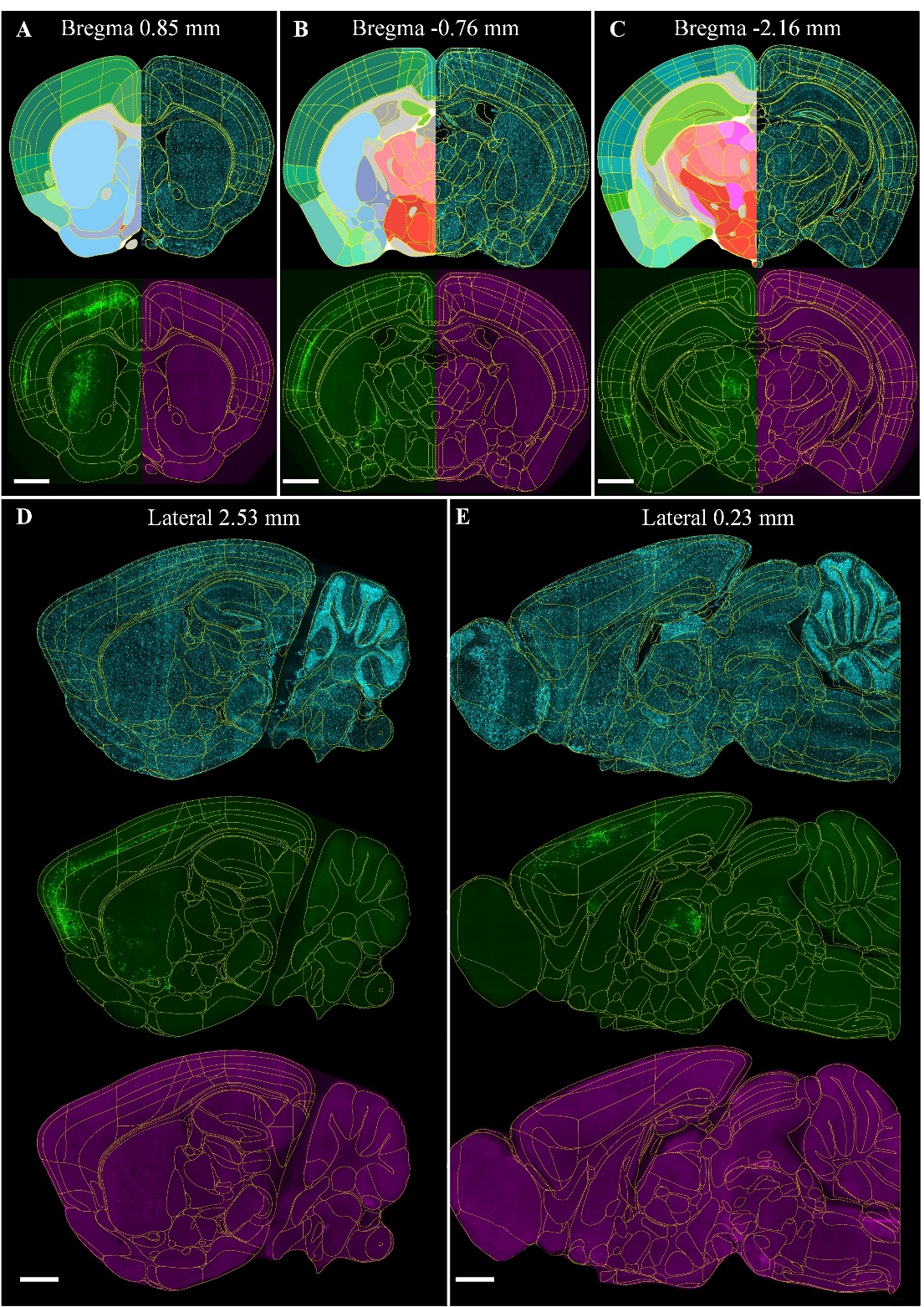


**Supplementary Figure 3.** Whole-brain registration by the BrainsMapi method. **(A-C)** The registration results of three typical coronal sections. The left and right halves of the image in the upper row show the Allen standard images and corresponding registered images in the blue channel. The left and right halves of the image in the lower row show the registered images in the green and red channels, respectively. **(D-E)** The registration results of two typical sagittal sections. The images in the upper, middle and lower rows show the registered images in the blue, green, and red channels, respectively. The projection thickness of all images was 10 μm. All images were superposed with the dotted line of the brain region contour extracted from the Allen standard atlas. Scale bars: 1 mm.


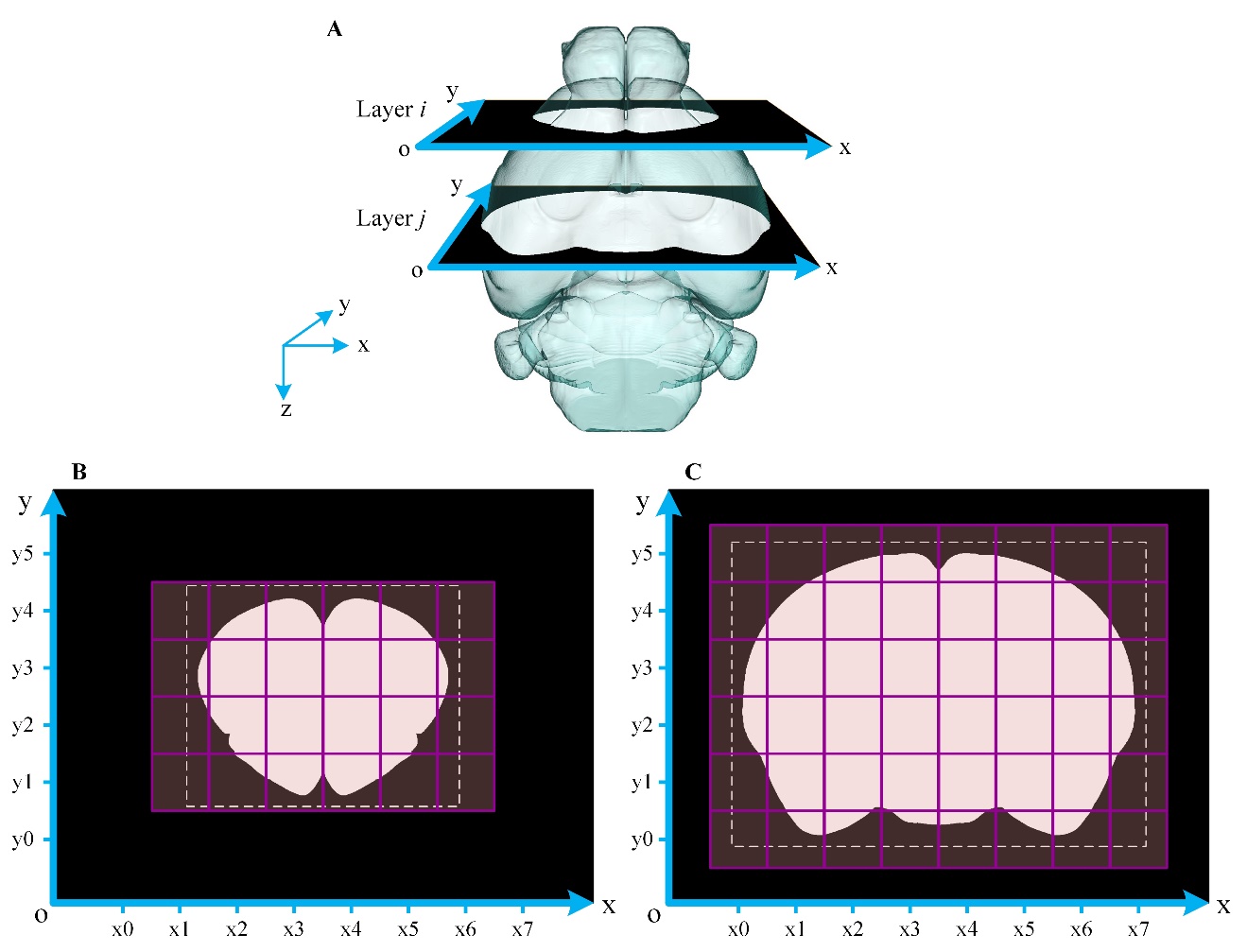


**Supplementary Figure 4.** Schematic diagram for automatically adjusting the actual imaging regions. **(A)** 3D rendering of the whole brain. Two coronal planes, Layer *i* and Layer *j*, indicate the corresponding positions of **(B-C)**, respectively. **(B-C)** Automatically recognition and definition of the imaging range of next layer. White dotted boxes indicated the minimum rectangular imaging regions predicted by automatic contour recognition. Brown regions with magenta boxes indicate the actual imaging region of the next layer. Each magenta square indicates single FOV. Image “Layer *i*” and Image “Layer *j*” are recorded at the same coordinates to ensure the auto-registration of the whole dataset.

# Supplementary Tables

**Supplementary Table 1.** Comparison of the number of imaged mosaics of imaging the whole mice brain using the multicolor WVT system with and without automatic contour recognition

| Sample No. | Number of layers | Number of the imaged mosaics in each channel | | Reduced ratio of the imaged mosaics (%) |
| --- | --- | --- | --- | --- |
|  |  | Without automatic contour recognition | With automatic contour recognition |  |
| 1 | 5,130 | 959,310 | 566,960 | 41 |
| 2 | 5,560 | 889,600 | 567,620 | 36 |
| 3 | 5,030 | 804,800 | 553,040 | 37 |
| 4 | 5,280 | 844,800 | 571,950 | 32 |
| 5 | 5,140 | 848,100 | 524,930 | 38 |
| 6 | 5,370 | 886,050 | 517,430 | 42 |
| 7 | 5,700 | 912,000 | 557,710 | 39 |
| 8 | 5,380 | 724,950 | 510,840 | 30 |
| Mean value | 5,324 | 858,701 | 546,310 | 37 |

**Supplementary Table 2.** Abbreviations for brain areas

| Anterior amygdalar area | AAA |
| --- | --- |
| Anterior cingulate area, dorsal part | ACAd |
| Nucleus accumbens | ACB |
| Accessory trigeminal nucleus | Acs5 |
| Agranular insular area, dorsal part | AId |
| Agranular insular area, posterior part | AIp |
| Agranular insular area, ventral part | AIv |
| Auditory areas | AUD |
| Basolateral amygdalar nucleus | BLA |
| Basomedial amygdalar nucleus | BMA |
| Bed nuclei of the stria terminalis | BST |
| Cerebellum | CB |
| corpus callosum | cc |
| Central amygdalar nucleus | CEA |
| central medial nucleus of the thalamus | CM |
| Caudoputamen | CP |
| Cortical Subplate | CTXsp |
| Undefined area in Cortical Subplate | CTXsp_0 |
| Dorsal column nuclei | DCN |
| Dorsal motor nucleus of the vagus nerve | DMX |
| Dorsal raphé | DR |
| Ectorhinal area | ECT |
| Entorhinal area, lateral part | ENTl |
| Endopiriform nucleus | EP |
| Frontal pole, cerebral cortex | FRP |
| Fundus of striatum | FS |
| External segment | GPe |
| Internal segment | GPi |
| Gigantocellular reticular nucleus | GRN |
| Gustatory areas | GU |
| Hindbrain | HB |
| Hippocampal formation | HPF |
| Hypothalamus | HY |
| Undefined area in Hypothalamus | HY_0 |
| intermediodorsal nucleus of the thalamus | IMD |
| Intermediate reticular nucleus | IRN |
| Lateral amygdalar nucleus | LA |
| Laterodorsal tegmental nucleus | LDT |
| lateral habenula | LH |
| Lateral hypothalamic area | LHA |
| Lateral preoptic area | LPO |
| Magnocellular nucleus | MA |
| Midbrain | MB |
| Undefined area in Midbrain | MB_0 |
| mediodorsal nucleus of the thalamus | MD |
| Medullary reticular nucleus | MDRN |
| Medial amygdalar nucleus | MEA |
| medial habenula | MH |
| Somatomotor areas | MO |
| Primary motor area | MOp |
| Secondary motor area | MOs |
| Medial preoptic area | MPO |
| Midbrain reticular nucleus | MRN |
| Undefined area in Medulla | MY_0 |
| Diagonal band nucleus | NDB |
| Nucleus of the solitary tract | NTS |
| Olfactory areas | OLF |
| Undefined area in Olfactory areas | OLF_0 |
| Orbital area, lateral part | ORBl |
| Orbital area, ventrolateral part | ORBvl |
| Olfactory tubercle | OT |
| Undefined area in Pons | P_0 |
| Periaqueductal gray | PAG |
| Pallidum | PAL |
| Undefined area in Pallidum | PAL_0 |
| Parvicellular reticular nucleus | PARN |
| Parabrachial nucleus | PB |
| parafascicular nucleus | PF |
| Paraflocculus | PFL |
| Pontine gray | PG |
| Paragigantocellular reticular nucleus | PGRN |
| Posterior hypothalamic nucleus | PH |
| Perihypoglossal nuclei | PHY |
| Piriform area | PIR |
| Prelimbic area | PL |
| Pedunculopontine nucleus | PPN |
| Pontine reticular nucleus, caudal part | PRNc |
| Pontine reticular nucleus | PRNr |
| Parasubthalamic nucleus | PSTN |
| Red nucleus | RN |
| Superior Colliculus, motor related | SCm |
| Substantia innominata | SI |
| Substantia nigra | SN |
| Substantia nigra, compact part | SNc |
| Substantia nigra, reticular part | SNr |
| Superior olivary complex | SOC |
| Somatosensory areas | SS |
| Primary somatosensory area | SSp |
| Primary somatosensory area, barrel field | SSp-bfd |
| Primary somatosensory area, lower limb | SSp-ll |
| Primary somatosensory area, mouth | SSp-m |
| Primary somatosensory area, nose | SSp-n |
| Primary somatosensory area, upper limb | SSp-ul |
| Primary somatosensory area, unassigned | SSp-un |
| Supplemental somatosensory area | SSs |
| Subthalamic nucleus | STN |
| Striatum | STR |
| Undefined area in Striatum | STR_0 |
| Temporal association areas | TEa |
| Thalamus | TH |
| Undefined area in Thalamus | TH_0 |
| Tegmental reticular nucleus | TRN |
| Motor nucleus of trigeminal | V |
| Facial motor nucleus | VII |
| facial nerve | VIIn |
| Anterior visual area | VISa |
| Visceral area | VISC |
| Primary visual area | VISp |
| Vestibular nuclei | VNC |
| ventral posterolateral nucleus of the thalamus, principal part | VPL |
| Ventral tegmental area | VTA |
| Hypoglossal nucleus | XII |
| Zona incerta | ZI |
